# Impact of Tumor *LINE-1* Methylation Level and Neoadjuvant Treatment and Its Association with Colorectal Cancer Survival

**DOI:** 10.3390/jpm10040219

**Published:** 2020-11-11

**Authors:** Hatim Boughanem, Gracia María Martin-Nuñez, Esperanza Torres, Isabel Arranz-Salas, Julia Alcaide, Sonsoles Morcillo, Francisco J Tinahones, Ana B Crujeiras, Manuel Macias-Gonzalez

**Affiliations:** 1 Department of Endocrinology and Nutrition, Virgen de la Victoria University Hospital, Institute of Biomedical Research in Malaga (IBIMA), University of Malaga, 29016 Malaga, Spain; h.b.boughanem@gmail.com (H.B.); graciamaria_mn@hotmail.com (G.M.M.-N.); sonsoles75@gmail.com (S.M.); mmacias.manuel@gmail.com (M.M.-G.); 2 CIBER in Physiopathology of Obesity and Nutrition (CIBEROBN), Instituto de Salud Carlos III, 28029 Madrid, Spain; anabelencrujeiras@hotmail.com; 3 Unidad de Gestión Clínica de Oncología Intercentros Hospital Universitario Virgen de la Victoria, 29010 Málaga, Spain; espe_torres2001@yahoo.es; 4 UGC de Anatomía Patológica, Hospital Universitario Virgen de la Victoria, 29010 Málaga, Spain; isabellanz@yahoo.es; 5 Medical Oncology Service, Hospital Costa del Sol, Red de Investigación en Servicios de Salud en Enfermedades Crónicas (REDISSEC), 29603 Marbella, Spain; drayulia@hotmail.com; 6 Epigenomics in Endocrinology and Nutrition Group, Instituto de Investigación Sanitaria (IDIS), Complejo Hospitalario Universitario de Santiago (CHUS/SERGAS), 15706 Santiago de Compostela, Spain

**Keywords:** *LINE-1*, methylation, tumor, survival, colorectal cancer

## Abstract

Recent studies suggest that long-interspersed nucleotide element-1 (*LINE-1*) hypomethylation is commonly found in colorectal cancer (CRC), and is associated with worse prognosis. However, the utility of *LINE-1* methylation on the prognosis of CRC is still controversial, and may be due to the fact that some clinical and pathological features may affect *LINE-1* methylation. Thus, the aim of this study was to assess the prognostic value of tumor *LINE-1* methylation in CRC, through their association with the CRC clinical and pathological characteristics. Survival of sixty-seven CRC patients was evaluated according to the median of tumor *LINE-1* methylation, as well as pathological and oncological variables. We also studied the association between *LINE-1* methylation and pathological features, and finally, we assessed the overall and disease-free survival of *LINE1* methylation, stratified by neoadjuvant treatment and further checked by multivariate Cox regression to assess the statistical interactions. *LINE-1* was hypomethylated in the CRC tumor with respect to the tumor adjacent-free area (*p* < 0.05), without association with any other clinical and oncological features, nor with overall and disease-free survival rates for CRC. Relevantly, in neoadjuvant treatment, *LINE-1* methylation was associated with survival rates. Thus, disease-free and overall survival rates of treated CRC patients were worse in the hypomethylated *LINE-1* tumors than those with normal *LINE-1* methylation (*p* = 0.004 and 0.0049, respectively). Indeed, *LINE-1* was hypermethylated more in the treated patients than in the non-treated patients (*p* < 0.05). The present study showed that tumor *LINE-1* hypomethylation was associated with worse survival rates in only treated patients. Our data suggest an interactive effect of neoadjuvant treatment and tumor *LINE-1* methylation, which could be a specific-tissue biomarker to predict survival of the treated patients, and help to personalize treatment in CRC.

## 1. Introduction

There is growing concern that colorectal cancer (CRC) will be the most frequent neoplasia of the 21th century [1]. In 2018, there was a worldwide estimate of 18.1 million new cancer cases and 9.6 million cancer deaths, which CRC accounts for approximately 6.1% for incidence and 9.2% for mortality, for both sexes combined [2]. Therefore, the screening tests need to achieve high accuracy using new prognostic and predictive biomarkers in order to establish more personalized strategies in the prevention of CRC. In this context, epigenetic modifications, and in particular, DNA methylation, have been widely investigated in a variety of prognostic applications in cancer [3,4]. Cancer prevention through epigenetic biomarkers is of particular interest to the clinical practice, due to their specificity and the diagnostic capability. Moreover, methylation of specific tumor suppressor genes has emerged as a useful approach in clinical practice and been proposed by multiple studies as potential biomarkers [5,6,7]. However, epigenetic biomarkers have been increasingly discussed in the literature, without standardized solutions or clinical validation yet, possibly due to the lack of adjusting for cofounding variables and pathological features that affect data reproducibility.

The methylation of long-interspersed nucleotide element (*LINE-1*) is commonly used as a marker of global DNA methylation [8]. *LINE-1* consist of retrotransposon elements located around the human genome [9]. In healthy tissue, *LINE-1* is found hypermethylated and inactivated. However, in cancer tissue, the methylation of *LINE-1* is typically found decreased and highly expressed, which is associated with increased retrotransposon activity [10]. Therefore, *LINE-1* can contribute to transcriptional disruptions and introduce genomic instability, which are the hallmarks of cancer [11]. Currently, *LINE1* is tightly linked to CRC, thus it is found progressively hypomethylated in the CRC tumor and associated with an increased risk of CRC [12,13]. *LINE-1* hypomethylation occurs during the early process of colorectal carcinogenesis, through normal colorectal mucosa to formation of adenoma and carcinoma [14,15]. Therefore, there are increasing bodies of work that support the idea that *LINE-1* hypomethylation can be used as prognostic biomarkers and therapeutic targets.

In the current literature, however, several studies found promising, but inconsistent, results about the prognostic value of *LINE-1* methylation in CRC. In this regard, a previous study pointed out that hypomethylation of tumor *LINE-1* had a significantly worse outcome overall in CRC, although no significant effect was observed on disease-free and recurrence-free survival [16]. Otherwise, a further study did not find an association between *LINE-1* methylation and overall survival [17]. In addition, a meta-analysis reported that *LINE-1* was significantly associated with the overall survival of CRC patients, which could be a predictive factor for CRC prognosis [18]. Subsequently, another study found significantly worse overall survival in the *LINE-1* hypomethylated patients in univariate cox analysis, but this association disappeared in the multivariate analysis [19]. Thus, it is worth noting though, that these results reflect a promising role of *LINE-1* in the prognosis of CRC. More work is needed to ensure that these associations, when considered, meet the clinical, pathological, and oncological outcomes in the management of the prognosis of CRC. 

We therefore hypothesized that the conflicting findings, observed in previous studies, might be explained, at least in part, by specific pathological and oncological variables, that could affect *LINE-1* methylation. This study aimed to investigate the association between tumor *LINE-1* methylation level and its relationship with clinical, pathological, and oncological features of CRC, to determine the potential role of *LINE-1* in the prognosis of CRC. This insight could offer a better understanding of the prognostic value of tumor *LINE-1* methylation in the prognosis of CRC.

## 2. Materials and Methods

### 2.1. Participants and Study Design

Sixty-seven CRC patients, who underwent surgery with curative intention, were recruited from the “Virgen de la Victoria” University Hospital (Málaga, Spain) between 2011 and 2013. All CRC patients were diagnosed by a pathological specialist, using biopsy and/or colonoscopy, whose medical records/pathological examinations were complete. Biopsy samples were classified according to the histological features by pathologists, and to the Classification of the “World Health Organization Classification of tumors of the Digestive System” (2016) [20]. The CRC patients were classified in two groups, 34 of whom had low *LINE-1* methylation, and, of them, were high *LINE-1* methylation according to cut-off of the median value of *LINE-1* methylation in tumor. Patients were treated according to standard protocols, by neoadjuvant treatment if indicated. This consisted of radiation therapy (total dose of 50 Gy delivered in 25 fractions of 2 Gy/fraction) and concomitant chemotherapy based on fluoropyrimidine. In all rectal cases, this included a total mesorectal excision, preceded. Follow-up was carried out according to local protocols, every three months in the first two years, and every six months from the third year. At each follow-up, a biochemical test, carcinoembryonic antigen (CEA), and physical examination were performed, and annual tests included colonoscopy. The exclusion criteria were patients with inflammatory and cardiovascular diseases, hereditary non-polyposis CRC or familial adenomatous polyposis, type 2 diabetes, insulin resistance, or renal and infectious diseases. We also excluded patients who had taken treatment that alters the lipid or glucose metabolism or who consumed >20 g of ethanol/day. Written informed consent was obtained from all patients and subjects and was reviewed and approved by the Ethics Committees of “Virgen de la Victoria” University Hospital (Málaga, Spain) (Registration number 0311/PI7).

### 2.2. Measurement of Biochemical Variables

Serum samples were obtained from blood samples by centrifugation for 15 min at 4000 rpm at 4 °C. Fasting glucose, total cholesterol, triglycerides, and high-density lipoprotein cholesterol (HDL-C) was obtained using Dimension Autoanalyzer (Dade Behring Inc., Deerfield, IL, USA). Low-density lipoprotein cholesterol (LDL-C) was calculated by the Friedewald equation [21]. Insulin level was carried out by radioimmunoassay methods using BioSource International Inc. (Camarillo, CA, USA). The homeostasis model assessment of insulin resistance (HOMA-IR) was calculated using the following equation: HOMA-IR = fasting insulin (μIU/mL) × fasting glucose (mmol/L)/22.5 [22]. CEA and carbohydrate antigen 19.9 (CA19.9) were measured by ELISA (DRG diagnostics, Germany).

### 2.3. DNA Extraction, Bisulfite Reaction, and Pyrosequencing for Methylation Analysis

Tumor samples and adjacent tumor-free samples were fixed using paraffin. DNA extraction was carried out by 10 sections of 14 µm from the tumor area and the adjacent tumor-free area. DNA from paraffin samples from the tumor area and the tumor-free area were obtained using Qiamp DNA FFPE (Formalin-Fixed Paraffin-Embedded) Tissue Kit following the instructions of the manufacturer (Qiagen GmbH, Hilden, Germany), with a xylene wash, to remove the paraffin. The purified DNA (2 μg) was used for bisulfite reaction using EpiTect Fast Bisulfite Kit (Qiagen GmbH, Hilden, Germany). The primer sequences and data about CpG sites for *LINE-1* are detailed in the Table S1. PCR reaction was performed using 0.2 nmol/L of primers. DNA pyrosequencing was carried out using the PyroMark Q96 ID pyrosequencing System (Qiagen GmbH, Hilden, Germany). The methylation average was presented as the percentage of methylated cytosine over the sum of methylated and unmethylated cytosines. Interassay precision (% CV) was 2.5% and intraassay (% CV) was 1.0%. Non-CpG cytosine residues were used as built-in controls to verify bisulfite conversion. We also used unmethylated and methylated DNA as controls in our assay (New England Biolabs, UK).

### 2.4. Statistical Analysis

The results are presented as mean ± standard deviation (SD) for continuous variables and as number (percentages) for categorical variables. Student *t*-test or Wilcoxon test was applied according to the normality of the variables. Pearson correlation coefficients between methylation and anthropometric and biochemical parameters and multivariate linear regression were performed. Kaplan–Meier curves were used for overall survival analyses. Hazard ratio (HR) was performed using multivariate Cox proportional hazards regression for *LINE-1* methylation. Odds ratio (OR) (95% confidence intervals (CIs)) was obtained by logistic regression analysis, taking low and high *LINE-1* methylation as a binary dependent variable. Analyses and graphic representation were pointed out, performed using R v.3.5.1 software (Integrated Development for R. RStudio, PBC, Boston, MA, USA), and significance *p* value was set at *p* < 0.05.

## 3. Results

### 3.1. Tumor LINE-1 Methylation Level and General Clinical and Pathological Characteristic Data of the Participants

The anthropometric and biochemical data from CRC patients with low (*n* = 34) and high *LINE-1* methylation (*n* = 33) are summarized in Table 1. No significant differences were observed between the anthropometric and biochemical variables of the CRC patients according to the *LINE-1* methylation status. There were no significant differences between low- and high-tumor *LINE-1* methylation groups in the tumoral markers, as CEA and CA19.9. The mean of *LINE-1* methylation value in the low and high *LINE-1* methylation groups were 52.41% (4.81) and 61.98% (3.21), respectively. As shown in Figure S1a, *LINE-1* was hypomethylated in the CRC tumor area versus the adjacent tumor-free area (*p* < 0.05). Table S1 summarizes clinical and oncological features, including location, stages, lymph node, and vascular invasion, as well as the presence of metastasis and neoadjuvant treatment, according to the tumor *LINE-1* methylation level. 

### 3.2. Pathological and Oncological Data, Tumor LINE-1 Methylation and Colorectal Cancer Patient Survival

We examined the relationship between the pathological features and overall and disease-free survival rates. We performed a cox regression model that includes CRC location (rectum vs. colon), stage (I + II vs. III + IV), lymph node and vascular invasion, metastasis, neoadjuvant treatment, and *LINE-1* methylation (Table 2). Tumor *LINE-1* methylation (low vs. high) did not reach statistical significance either with overall (univariate: HR: 0.71; 95% CI: 0.28–1.76; multivariate: HR: 0.95; 95% CI: 0.28–3.20) or disease-free survival (univariate: HR: 0.9; 95% CI: 0.37–2.2; multivariate: HR: 1.29; 95% CI: 0.42–3.9) in CRC patients. However, only metastasis was an independent prognostic factor for CRC patients, including overall (univariate: HR: 5.22; 95% CI: 2.11–12.86; multivariate: HR: 8.65; 95% CI: 1.54–45.31) and disease-free survival rates (univariate: HR: 8.9; 95% CI: 3.4–24.0) multivariate: HR: 8.99; 95% CI: 1.74–46.4), which was associated with an increased risk of worse survival rates.

### 3.3. Tumor LINE-1 Methylation and Colorectal Cancer Patient Survival in Strata of Neoadjuvant Treatment

In the secondary analysis, we conducted an exploratory analysis to examine the relationship between *LINE-1* methylation and CRC pathological features, according to a linear and logistic (low vs. high) regression analysis. In the multivariate linear regression analysis, it was observed that location, metastasis, and neoadjuvant treatment could explain the variation of *LINE-1* methylation level, in a regression model, that included stage, lymph node, and vascular invasion (Table 3). Furthermore, we performed a logistic regression analysis to determine what factors could predict the variation of *LINE-1* methylation. We observed that location did not reach significant statistical value. However, metastasis (OR: 0.55; 95% CI: 0.33–0.89) and neoadjuvant treatment (OR: 1.91; 95% CI: 1.43–2.56) maintained significant statistical values (*p* < 0.05). 

**Figure 1:**
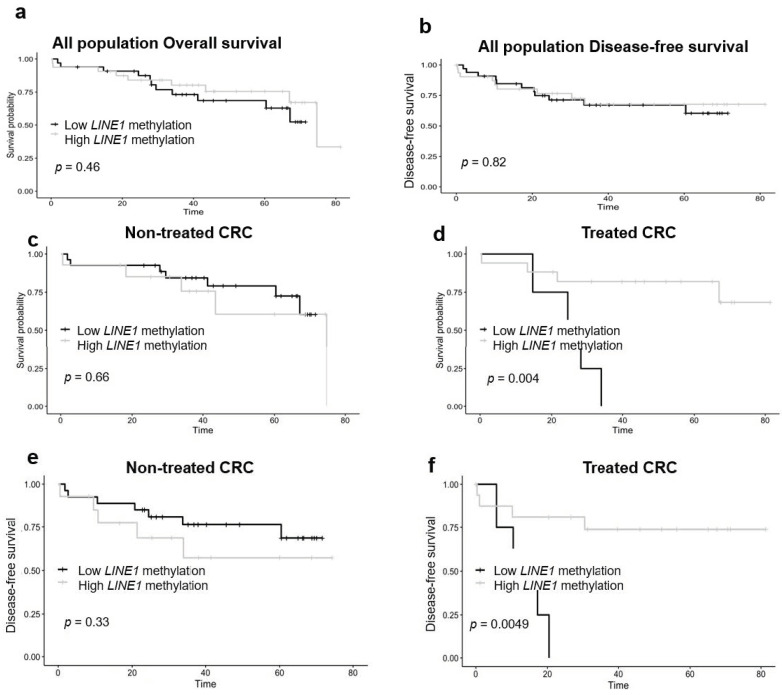
*LINE-1* methylation and overall survival analysis according to the neoadjuvant treatment. Kaplan–Meier analysis was performed to determine overall and disease-free survival according *LINE-1* methylation (low vs. high) of overall survival and disease-free survival of all populations (**a**,**b**), and non-treated (**c**,**e**) and treated patients (**d**,**f**). Significant differences are conducted according to Wald test *(p* < 0.05). CRC—colorectal cancer.

In the Kaplan–Meier analysis for overall and disease-free survival (Figure 1), tumor *LINE-1* methylation (low vs. high) was not associated with worse overall and disease-free survival in overall population (Figure 1a,b) and when stratifying by metastasis (Figure S2). When stratifying by neoadjuvant treatment, non-treated CRC patients did not reach significant statistical results for overall and disease-free survival (Figure 1c,e). However, CRC treated patients reached significant statistical results. The low *LINE-1* methylation group had a worse overall (*p* = 0.004) and disease-free survival rate (*p* = 0.005) than the high *LINE-1* methylation group, being that the medians of overall and disease-free survival were 25.34 and 13.45 months, respectively (Figure 1d,f). Finally, *LINE-1* methylation was hypermethylated in treated patients in comparison with non-treated patients (Figure S1b) (*p* < 0.05).

## 4. Discussion

In this study, we hypothesized that tumor *LINE-1* methylation might be related with different pathological features and further act as a possible prognostic factor for CRC outcomes. Utilizing an overview of low and high *LINE-1* methylation, we found for the first time a statistically significant association between tumor *LINE-1* methylation and neoadjuvant treatment in CRC patients, which was related with survival outcomes. Relevantly, only in treated patients, tumor *LINE-1* hypomethylation was associated with worse overall and disease-free survival in those with tumor *LINE-1* hypermethylation. The prognostic utility of tumor *LINE-1* methylation in CRC-treated patients could serve as a specific-tissue biomarker to predict survival of the treated patients, which could help to personalize treatment in CRC patients. 

Currently, several studies have considered tumor *LINE-1* methylation in survival analysis, but conflicting results about the survival rate of CRC and *LINE-1* methylation were observed. A previous study reported that *LINE-1* hypomethylated tumors had a significantly worse overall survival than in the *LINE-1* hypermethylated tumors only in proximal colon cancers, but not in distal colon and rectum cancer, suggesting an interactive effect of *LINE-1* methylation level and CRC location [23]. A study observed that overall survival, but not disease-free and recurrence-free survival, was worse in the tumor *LINE-1* hypomethylation patients [16], although another did not find an association between *LINE-1* methylation and overall survival [18]. In addition, worse overall survival was observed in the tumor *LINE-1* methylated patients in a previous study, but this association disappears in the multivariate analysis [19]. Overall, none of the previous studies found a consensus of the prognostic value of tumor *LINE-1* methylation on the survival outcome, whose results were limited and did not examine the interactive association of some cofounding variables, such as location or neoadjuvant treatment [24,25]. In our results, we did not find association between tumor *LINE-1* methylation and overall and disease-free survival in CRC patients. We also did not find significant value by studying stage (I + II vs. III + IV) on overall and disease-free survival, although significance was detected in metastasis. This is because, in our cohort, all metastatic patients were in stage IV, but not in stage III, which could explain that the stage stratification did not reach significance. However, *LINE-1* methylation was associated with some pathological variables, such as location, metastasis, and neoadjuvant treatment, which could affect the variation of *LINE-1* methylation. Indeed, metastasis was an independent prognostic factor risk to predict overall and disease-free survival, which was in line with the literature [26].

Our results also showed that tumor *LINE-1* methylation was associated with location in the linear regression model, although the logistic model did not reach a significant statistical value. In this sense, a study showed that *LINE-1* hypomethylated tumors had a significantly worse overall survival than in the *LINE-1* hypermethylated tumors only in proximal colon, but not in rectum cancer [23], suggesting that a local action of *LINE-1* must be considered in CRC. Furthermore, additional studies in large cohorts, and future challenges considering location and neoadjuvant treatment, do not concern only achieving high accuracy on the prognosis of CRC, but also selecting CRC patients who could show better benefits from the neoadjuvant treatment, based on the tumor *LINE-1* methylation.

Epigenetic alterations are promising biomarkers for cancer detection. DNA methylation of *LINE-1* methylation showed a strong association with CRC. Our results were in line with the literature, since *LINE-1* methylation is found progressively hypomethylated in CRC tumor and associated with an increased risk of CRC [12,13,14]. We also found that *LINE-1* in treated patients was hypomethylated in comparison with non-treated patients, suggesting that neoadjuvant treatment could be implicated in the variation of *LINE-1* methylation. *LINE-1* methylation also had more significant impact on survival analysis. We observed that the hypomethylation of *LINE-1* was significantly associated with worse overall and disease-free survival rates in only CRC treated patients. The prognostic utility of this finding could serve as a specific-tissue biomarker to predict survival rates in neoadjuvant-treated patients, and could purpose *LINE-1* as a biomarker to personalize treatment in CRC patients. This fact is relatively novel, although more studies are needed to confirm these observations, *LINE-1* methylation could be purposed as a potential biomarker to improve clinical response and monitor neoadjuvant therapy.

Our study has some limitations. Although the sample size used in this study seemed to be relatively small, our recruited model was based on interventional, as well as prospective–continuous observational studies. We also established a restricted inclusion and exclusion criteria, and all CRC patients were selected and further underwent surgery with curative intention. In addition, we examined the two pathological samples from specimen biopsy, using both the tumor tissue and adjacent tumor-free tissue from surgical patients. In further studies, a larger sample size and validation studies from other cohorts should be included to construct a prognostic model for monitoring the neoadjuvant therapy, as well as the clinical response for CRC outcomes.

## 5. Conclusions

In this study, we evaluated approaches to provide new applications for the *LINE-1* methylation in CRC prognosis. In spite of our results being only the initial step, our findings suggest that *LINE-1* methylation could be considered as a potential biomarker for predict survival rate in patients treated for CRC. Firstly, we showed that *LINE-1* was hypomethylated in the CRC tumor compared to the tumor adjacent-free area and was hypermethylated in the treated patients compared to the non-treated patients. However, *LINE-1* did not associate with survival rates, and only metastasis was significantly associated with patient’s outcome. Worse survival in tumor *LINE-1* hypomethylation was observed only in treated patients. Although these results should be interpreted with caution, our data suggest an interactive effect of neoadjuvant treatment and tumor *LINE-1* methylation on pathological and oncological CRC outcome.

## Figures and Tables

**Table 1:** Baseline characteristics of the epidemiological and clinical variables of the population study.

Variables	Low *LINE-1* Methylation (%)	High *LINE-1* Methylation (%)	*p*
*n*	34	33	
Age (years)	66.29 ± 10.75	67.27 ± 10.19	0.703
Sex (male/female)	24/10	21/12	0.544
BMI (kg/m^2^)	26.87 ± 4.37	27.05 ± 3.46	0.853
Glucose (mg/dL)	117.51 ± 34.94	124.63 ± 52.16	0.517
Insulin (µUI/mL)	5.54 ± 3.71	6.60 ± 5.57	0.380
HOMA-IR	1.68 ± 1.37	2.06 ± 2.08	0.383
Total cholesterol (mg/dL)	166.58 ± 41.02	177.27 ± 36.58	0.268
Triglycerides (mg/dL)	167.51 ± 97.85	162.42 ± 66.97	0.806
LDL (mg/dL)	99.11 ± 32.84	107.09 ± 29.64	0.978
HDL (mg/dL)	40.33 ± 11.44	40.24 ± 15.37	0.304
CEA (mg/dL)	7.27 ± 11.27	4.42 ± 9.62	0.297
CA19.9 (U/mL)	21.78 ± 28.24	21.93 ± 34.72	0.984

Data are expressed as mean ± standard deviations or frequency numbers. Significant differences between groups were performed according to Welch’s two sample tests (*p* < 0.05). Chi-squared test was used for variables expressed as frequency numbers (*p* < 0.05). Abbreviations: CRC: colorectal cancer; BMI: body mass index; HOMA-IR: homeostasis model assessment of insulin resistance; HDL high-density lipoprotein; LDL: low-density lipoprotein; CEA: carcinoembryonic antigen; CA19.9: cancer antigen type 19.9. *LINE-1*: long-interspersed nucleotide element 1.

**Table 2:** Univariate and multivariate analysis for colorectal cancer patient survival.

Variables	Overall Survival		Disease-Free Survival	
	Univariate HR (95% CI)	Multivariate HR (95% CI)	Univariate HR (95% CI)	Multivariate HR (95% CI)
Location (rectum vs. colon)	1.28 (0.57–2.90)	1.09 (0.29–4.18)	1.1 (0.48–2.50)	1.44 (0.37–5.4)
Stage (I + II vs. III + IV)	1.60 (0.62–3.84)	1.11 (0.09–13.72)	2.3 (0.97–5.4)	2.18 (0.20–24.0)
Lymph node (Negative vs. positive)	1.41 (0.58–3.43)	0.68 (0.07–6.44)	2.1 (0.86–4.9)	0.62 (0.07–5.7)
Vascular invasion (Negative vs. positive)	1.84 (0.71–4.79)	1.94 (0.58–6.45)	2.1 (0.82–5.5)	2.2 (0.67–7.4)
Metastasis (Negative vs. positive)	5.22 (2.11–12.86) *	8.65 (1.54–45.31) *	8.9 (3.4–24.0) *	8.99 (1.74–46.4) *
Neoadjuvant therapy (Negative vs. positive)	1.12 (0.48–2.64)	0.85 (0.20–3.70)	1.4 (0.6–3.3)	0.78 (0.18–3.3)
*LINE-1* methylation (Low vs. high)	0.71 (0.28–1.76)	0.95 (0.28–3.20)	0.9 (0.37–2.2)	1.29 (0.42–3.9)

Univariate and multivariable Cox regression model of overall and disease-free survival, and the follow-up time for all patients was 80 months. The bold number and asterisks indicated significant results, which *p* value was calculated by the Wald test. Abbreviations: *LINE-1*: long-interspersed nucleotide element 1; HR: hazard ratio; CI: confidence interval.

**Table 3:** The association between *LINE-1* methylation status and clinicopathological parameters.

Variables	*LINE-1* Methylation Levels (Continuous) β (SD)	*LINE-1* Methylation Levels (Low vs. High) OR (95% CI)
Location (rectum vs. colon)	3.26 (1.44) *	1.19 (0.91–1.56)
Stage (I + II vs. III + IV)	4.36 (3.60)	1.92 (0.97–3.77)
Lymph node (Negative vs. positive)	−2.27 (3.53)	0.55 (0.28–1.06)
Vascular invasion (Negative vs. positive)	−1.31 (1.65)	1.01 (0.74–1.38)
Metastasis (Negative vs. positive)	−6.54 (2.61) *	0.55 (0.33–0.89) *
Neoadjuvant treatment (Negative vs. positive)	8.65 (1.55) *	1.91 (1.43–2.56) *

The multivariate linear and logistic regression analyses were performed using *LINE-1* methylation as dependent variable, and location, stage, lymph node and vascular invasion, metastasis and neoadjuvant treatment as independent parameters. Bold numbers and asterisks indicate significant correlation (* *p* < 0.05).

## Supplementary Materials

The following are available online at https://www.mdpi.com/2075-4426/10/4/219/s1, Figure S1: LINE-1 methylation status according to the tumor-free and tumor state and between non-treated and treated patients, Table S1: Clinicopathological features of low and high LINE-1 methylation in tumor from colorectal cancer patients. Figure S2. LINE1 methylation and overall survival analysis according to metastasis. Table S2. Cox analysis for colorectal cancer patient survival according to *LINE-1* and adjusted variables.
