# Supplementary material for: Impact of Tumor LINE-1 Methylation Level and Neoadjuvant Treatment and Its Association with Colorectal Cancer Survival
Source: J Pers Med. 2020 Nov 11;10(4):219. doi: 10.3390/jpm10040219 (PMC7712476; doi:10.3390/jpm10040219)
Supplement: Supplementary file 1 [file jpm-10-00219-s001.zip › jpm-969431-supplementary.pptx]

## Slide 1
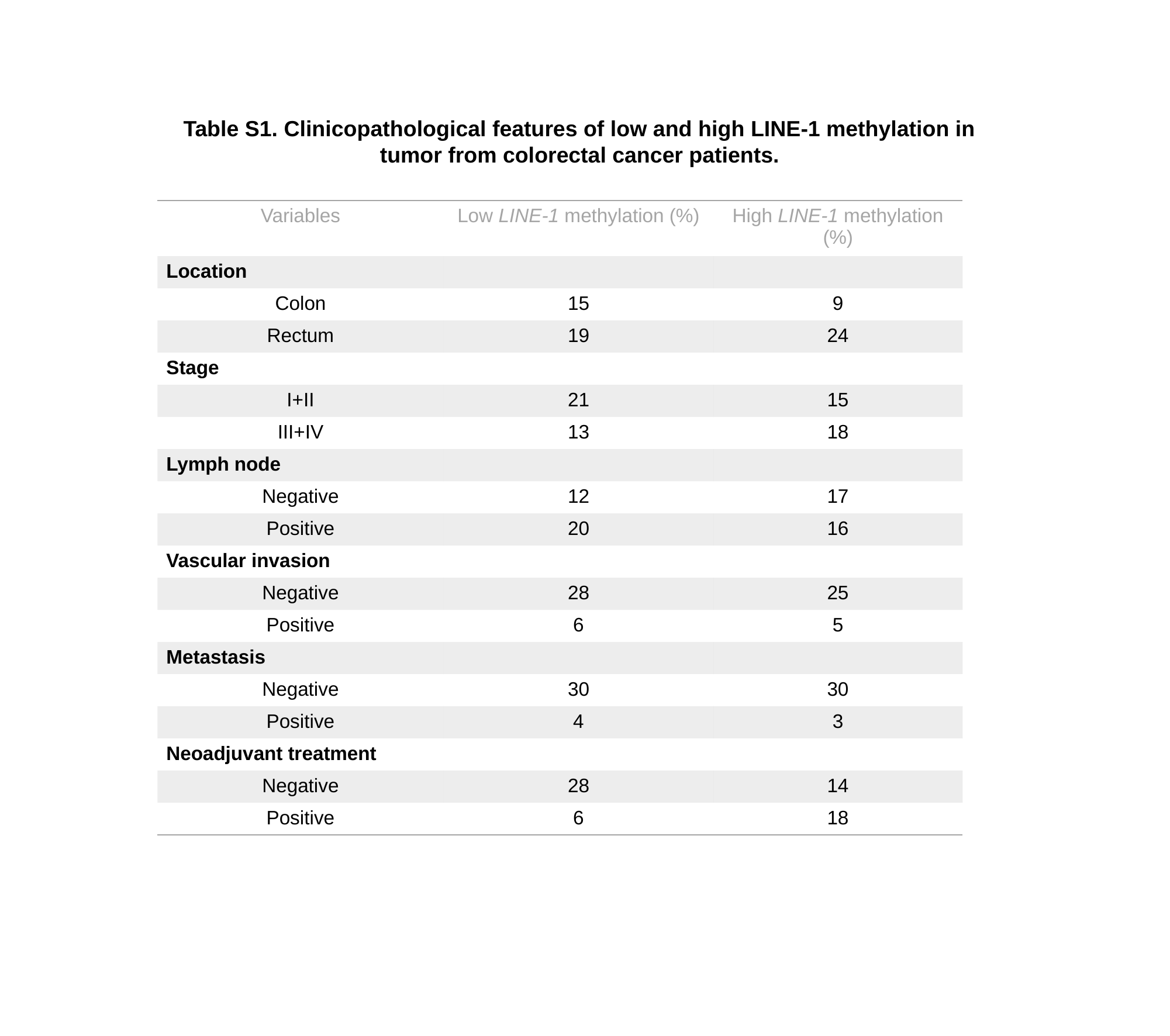

Table S1. Clinicopathological features of low and high LINE-1 methylation in tumor from colorectal cancer patients.
| Variables | Low LINE-1 methylation (%) | High LINE-1 methylation (%) |
| --- | --- | --- |
| Location | | |
| Colon | 15 | 9 |
| Rectum | 19 | 24 |
| Stage | | |
| I+II | 21 | 15 |
| III+IV | 13 | 18 |
| Lymph node | | |
| Negative | 12 | 17 |
| Positive | 20 | 16 |
| Vascular invasion | | |
| Negative | 28 | 25 |
| Positive | 6 | 5 |
| Metastasis | | |
| Negative | 30 | 30 |
| Positive | 4 | 3 |
| Neoadjuvant treatment | | |
| Negative | 28 | 14 |
| Positive | 6 | 18 |

## Slide 2
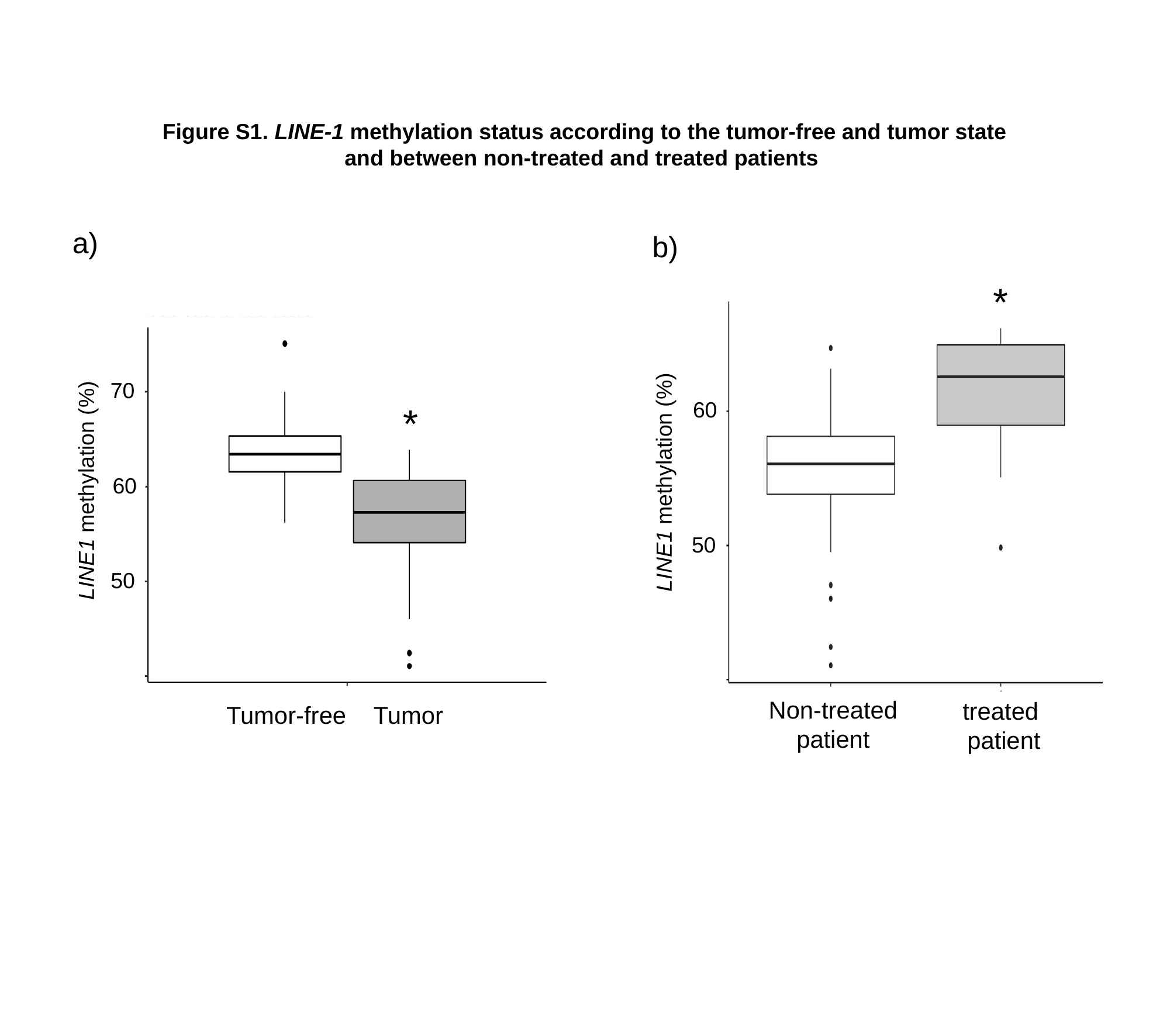

Figure S1. LINE-1 methylation status according to the tumor-free and tumor state and between non-treated and treated patients
a)
b)
*
*
Tumor-free
Tumor
70
60
LINE1 methylation (%)
60
LINE1 methylation (%)
50
50
Non-treated patient
treated
patient

## Slide 3
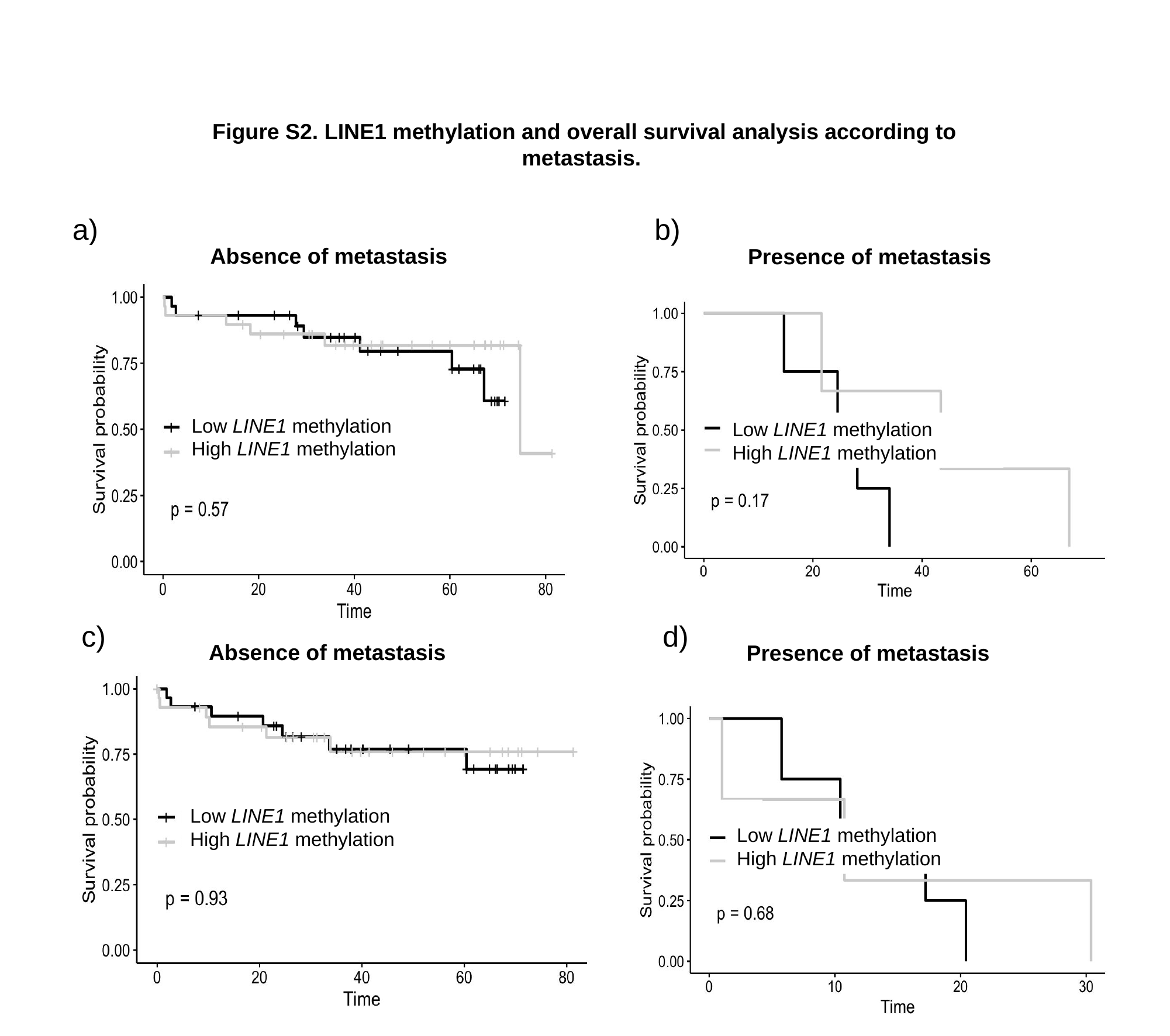

Figure S2. LINE1 methylation and overall survival analysis according to metastasis.
b)
a)
Absence of metastasis
Presence of metastasis
Low LINE1 methylation
High LINE1 methylation
Low LINE1 methylation
High LINE1 methylation
d)
c)
Absence of metastasis
Presence of metastasis
Low LINE1 methylation
High LINE1 methylation
Low LINE1 methylation
High LINE1 methylation

## Slide 4
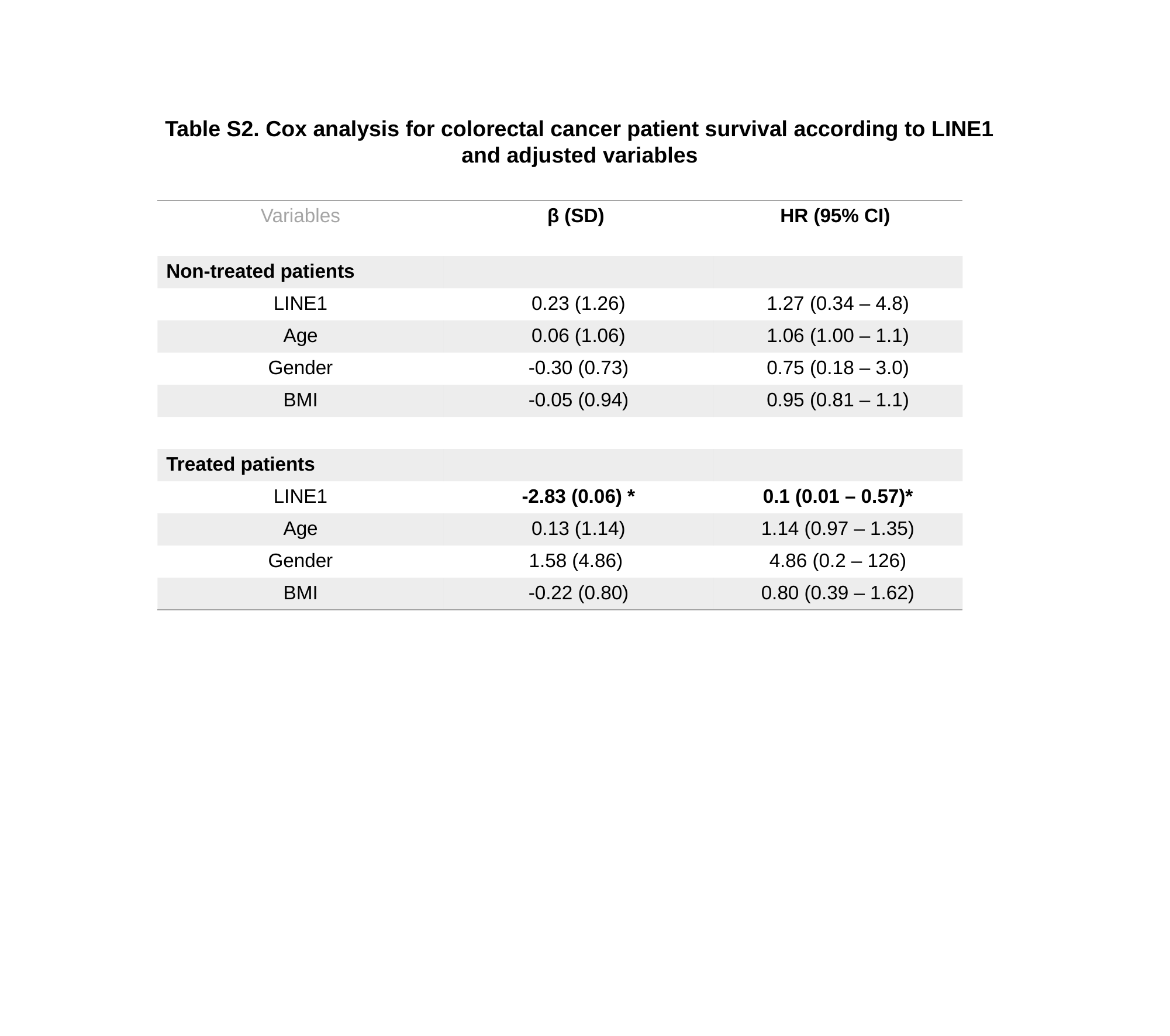

Table S2. Cox analysis for colorectal cancer patient survival according to LINE1 and adjusted variables
| Variables | β (SD) | HR (95% CI) |
| --- | --- | --- |
| Non-treated patients | | |
| LINE1 | 0.23 (1.26) | 1.27 (0.34 – 4.8) |
| Age | 0.06 (1.06) | 1.06 (1.00 – 1.1) |
| Gender | -0.30 (0.73) | 0.75 (0.18 – 3.0) |
| BMI | -0.05 (0.94) | 0.95 (0.81 – 1.1) |
| | | |
| Treated patients | | |
| LINE1 | -2.83 (0.06) \* | 0.1 (0.01 – 0.57)\* |
| Age | 0.13 (1.14) | 1.14 (0.97 – 1.35) |
| Gender | 1.58 (4.86) | 4.86 (0.2 – 126) |
| BMI | -0.22 (0.80) | 0.80 (0.39 – 1.62) |
